# Immune Response and Apoptosis-Related Pathways Induced by *Aeromonas schubertii* Infection of Hybrid Snakehead (*Channa maculata*♀ × *Channa argus*♂)

**DOI:** 10.3390/pathogens10080997

**Published:** 2021-08-07

**Authors:** Chun Liu, Jie Ma, Defeng Zhang, Wei Li, Biao Jiang, Zhendong Qin, Youlu Su, Li Lin, Qing Wang

**Affiliations:** 1 Guangzhou Key Laboratory of Aquatic Animal Diseases and Waterfowl Breeding, Innovative Institute of Animal Healthy Breeding, College of Animal Sciences and Technology, Zhongkai University of Agriculture and Engineering, Guangzhou 510225, China; Liuchun@zhku.edu.cn (C.L.); m320668996@163.com (J.M.); liwei@zhku.edu.cn (W.L.); Jiangbiao@zhku.edu.cn (B.J.); Qinzhendong@zhku.edu.cn (Z.Q.); 2 Key Laboratory of Aquatic Animal Immune Technology of Guangdong Province and Key Laboratory of Fishery Drug Development of Ministry of Agriculture, Pearl River Fisheries Research Institute, Chinese Academy of Fishery Sciences, Guangzhou 510380, China; zhangdefeng08@126.com

**Keywords:** hybrid snakehead (*Channa maculata*♀ × *Channa argus*♂), *Aeromonas schubertii*, RNA-seq, immune response, apoptosis

## Abstract

*Aeromonas schubertii* is the etiological pathogen of internal organ nodules in snakehead fish. Infections with *A. schubertii* produce a significant economic loss in aquaculture. Therefore, it is important to examine the immune mechanisms by which snakeheads defend against *A. schubertii* infection. In this study, we established a hybrid snakehead infection model by intraperitoneal injection of *A. schubertii* that produced internal organ nodules. The splenic immune response of infected fish was examined at the transcriptome level by Illumina-seq analysis. Results showed 14,796 differentially expressed genes (DEGs) following *A. schubertii* infection, including 4441 up-regulated unigenes and 10,355 down-regulated unigenes. KEGG analysis showed 2084 DEGs to be involved in 192 pathways, 14 of which were immune-related. Twelve DEGs were used to validate quantitative real-time PCR results with RNA-seq data. Time-course expression analysis of six genes demonstrated modulation of the snakehead immune response by *A. schubertii.* Furthermore, transcriptome analysis identified a substantial number of DEGs that were involved in the apoptosis signaling pathway. TUNEL analysis of infected spleens confirmed the presence of apoptotic cells. This study provided new information for a further understanding of the pathogenesis of *A. schubertii* in snakeheads, which can be used to prevent and possibly treat *A. schubertii* infections.

## 1. Introduction

*Channa argus* and *Channa maculata*, commonly known as the snakehead fishes, are native to most areas of China and belong to the family *Channidae*. Because of their delicious taste and traditional Chinese medicinal properties, snakeheads have for a long time been economically important and widely cultivated in ponds and reservoirs throughout China [1]. Hybrid snakehead (*C. maculata*♀ *× C. argus*♂) is the first filial generation of *C. argus* (male parent) and *C. maculata* (female parent). Due to the advantages of heterosis in commercial fish farming (high growth rate and strong disease resistance) these crossbred fish are a valuable commodity and are one of the most popular species cultivated in most areas of China. Snakehead production exceeds 510,000 tons (worth~1.6 billion US dollars) annually [2], with hybrid snakehead being the predominant fish.

With intensive aquaculture farming within the last few years, snakehead infections by *Aeromonas* *schubertii* have become significant. This bacterium belongs to the moderate Aeromonas complex group and is a Gram-negative rod present in freshwater, seawater, and soil [3]. It was first isolated in 1988 from human clinical specimens including abscesses, wounds, skin, pleural fluid, and blood [4]. Accumulated clinical evidence demonstrated *A. schubertii* to be involved in the etiology of a variety of human diseases such as skin and soft-tissue infections, diarrhea, and necrotizing fasciitis [5]. Recently, *A. schubertii* has been reported to be the causative agent for outbreaks of “white spots disease” in snakehead fish: *C. argus* [6], *C. maculata* [7], and the hybrid snakehead [8]. The typical symptoms of the disease are severe multiple white nodules scattered throughout the spleen, liver, and kidney [6,9]. The disease has a high mortality rate of approximately 45%, resulting in serious economic loss, which jeopardizes the development of snakehead aquaculture [6].

As in most microbial infections, hosts are highly dependent on the immune system for protection from infection. Teleosts are one of the earliest evolutionary groups with both innate and adaptive forms of immunity [10]. Numerous immune-related genes have been identified in teleosts [11]. In the past few years, transcriptome sequencing technology has been used to identify immune-related genes, which has substantially improved the understanding of the host immune defense against various pathogens [12]. For example, Li et al. used RNA-seq to analyze the mucosal immune response of *Ictalurus punctatus* and found signatures for intestinal barrier disruption and pathogen entry following *Edwardsiella ictaluri* infection [13]. Transcriptome analysis by Wang et al. identified changes in multiple immune-related genes in *Larimichthys crocea* during *Cryptocaryon irritans* infection [14]. Transcriptome profiling analysis has also been used for *Epinephelus coioides* [15], *Cynoglossus semilaevis* [16], and *Ctenopharyngodon idella* [17]. Although white spots disease, caused by *A. schubertii*, is one of the most serious diseases in snakehead fish [18], few reports have considered the host immune response to this pathogen during infection.

Apoptosis is considered a host immune defense mechanism [19,20,21]. Apoptotic death of infected cells is thought to limit the spread of pathogens by protecting the integrity of surrounding tissues [22,23,24]. In some infections, pathogenic bacteria can either induce or inhibit host cell apoptosis [25,26]. For example, bacteria-induced apoptosis promotes an inflammatory response that causes cell death and tissue damage, which furthers bacterial colonization [27,28]. In contrast, some bacteria inhibit apoptosis to prevent efferocytosis, thus evading innate host defense [29,30,31]. Therefore, apoptosis can play an important role in host-pathogen interactions [32]. In this study, we have focused on tissue apoptosis in the hybrid snakehead after *A. schubertii* infection.

In vivo transcriptome profiling of snakehead *A. schubertii* infection was performed by RNA sequencing. Apoptosis was demonstrated by a TUNEL assay. The expression patterns of several genes involved in the innate immune response pathway and the apoptotic pathway were confirmed by RT-qPCR. The purpose of this study was to systematically and comprehensively understand the molecular immune mechanisms and apoptosis-related pathways that underlie the host response to *A. schubertii*.

## 2. Results

### 2.1. Transcriptome Sequencing and De Novo Assembly

**Figure 1:**
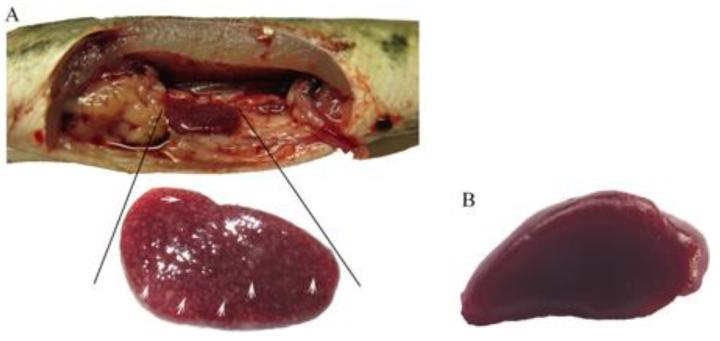
The *Aeromonas schubertii* infection model in hybrid snakehead. (**A**) White nodules (indicated by white arrows) were distributed in an infected spleen. (**B**) Spleen from the negative control.

**Figure 2:**
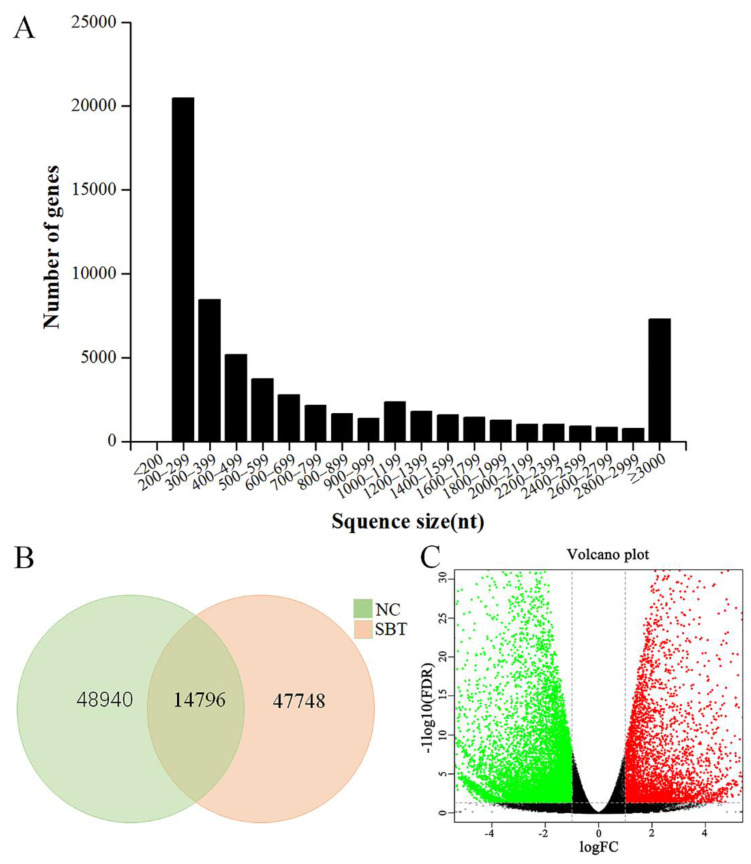
(**A**) Overview of *A. schubertii* infected hybrid snakehead transcriptome sequence length distribution for all unigenes. The *x*-axis indicates the interval length of the transcript and the *y*-axis indicates the number of transcripts with different lengths. (**B**) Venn diagram of control and *A. schubertii* intergroup expression. (**C**) Volcano plot of DEGs identified in NC and SBT spleens. Green and red dots indicate down-regulated and up-regulated DEGs, respectively.

For transcriptome sequencing, an infectious model of *A. schubertii* in juvenile hybrid snakehead was established by intraperitoneal injection of the pathogenic bacteria. Three days post-infection, numerous apparent white nodules, approximately 1.0–2.0 mm in diameter, were observed particularly on the spleen and on several other internal organs (Figure 1). Spleen samples were collected 8 h post-infection and used for transcriptomic sequencing. A total of 195,057,214 raw reads [103,354,170 and 91,703,044 reads generated from control (NC) and *A. schubertii* infected (SBT), respectively] were obtained. After cleaning of low-quality reads, there were 102,015,252 and 90,423,428 clean reads for the NC and SBT groups, respectively (Table 1). All cleaned data were assembled with Trinity software by de novo assembly, which generated a total of 65,884 unigenes, with a mean length of 1159 base pairs (bp). The range was from 201 to 26,244 bp with an N50 value of 2708 bp. Among these unigenes, 34,096 (51.75%) were 150–500 bp, 11,618 (17.63%) were 500–1000 bp, 8363 (12.69%) were 1000–2000 bp, with 11,807 (17.92%) over 2000 bp (Figure 2A). Levels of gene expression were standardized to RPKM. A total of 63,736 and 62,544 contigs were detected for NC and SBT, respectively, with 14,796 common contigs (Figure 2B). All unigenes were compared against protein databases including; the NCBI nonredundant protein database (Nr), the Swiss-Prot database, the KOG database, and the KEGG database by BLAST (Table 2).

### 2.2. Functional Explanation of Differentially Expressed Genes (DEGs)

To search DEGs from NC and SBT, the threshold was set for log_2_ (fold-change) | > 1 and *p*-value < 0.01. A total of 14,796 DEGs were identified, including 4441 (accounting for 30.01% of all DEGs) up-regulated genes and 10,355 (accounting for 69.99%) down-regulated genes. DEGs after *A schubertii* infection were visualized by volcano plot. Down-regulated DEGs (green dots) were greater than up-regulated DEGs (red dots) (Figure 2C).

**Figure 3:**
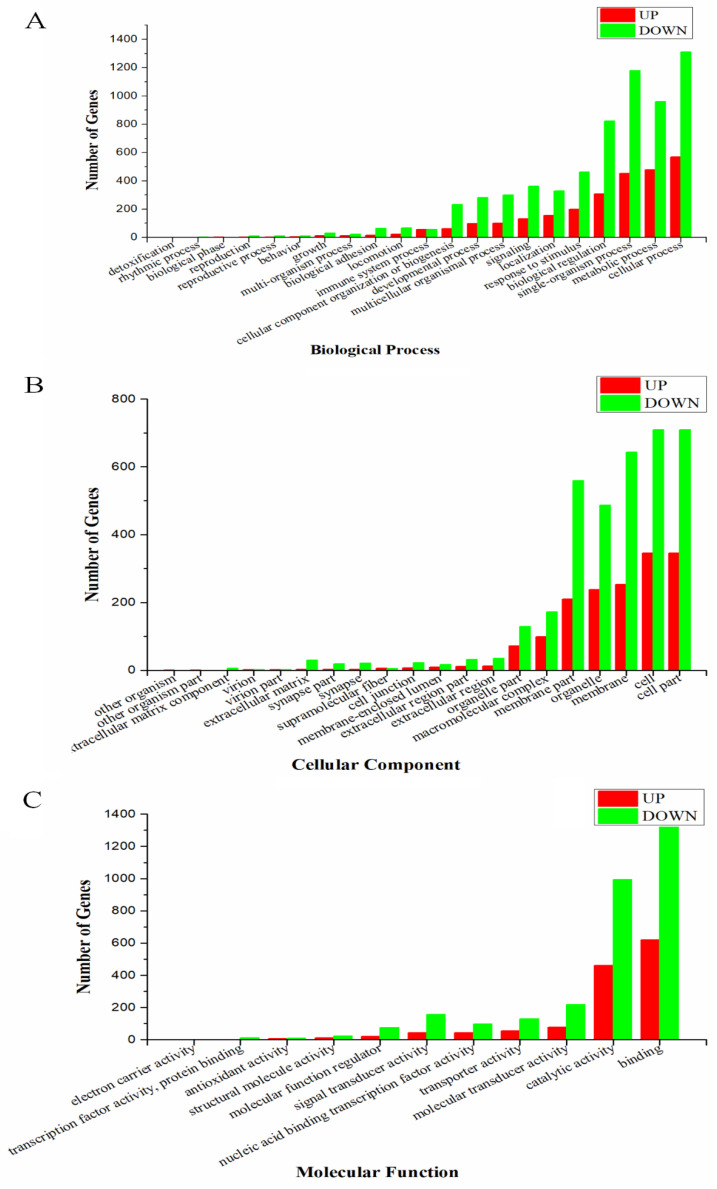
Gene ontology enrichment analysis of DEGs in *A. schubertii* infected hybrid snakehead. The differentially expressed genes (DEGs) were classified into three subclasses, including biological process (**A**), cellular component (**B**), and molecular function (**C**).

**Figure 4:**
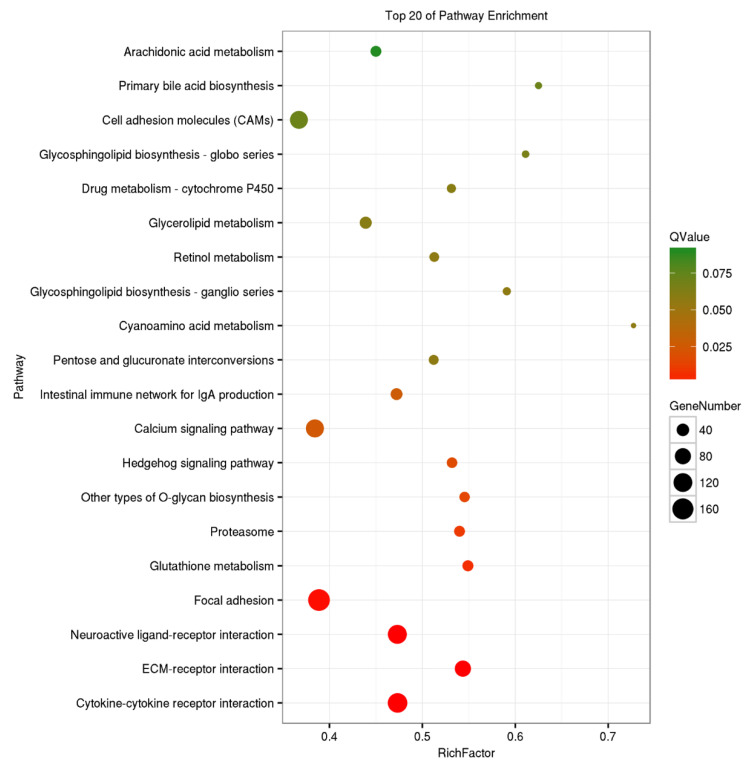
Scatter plots of the top 20 enriched KEGG pathways. The rich factor is the ratio of DEGs to all the genes annotated for this pathway term.

**Figure 5:**
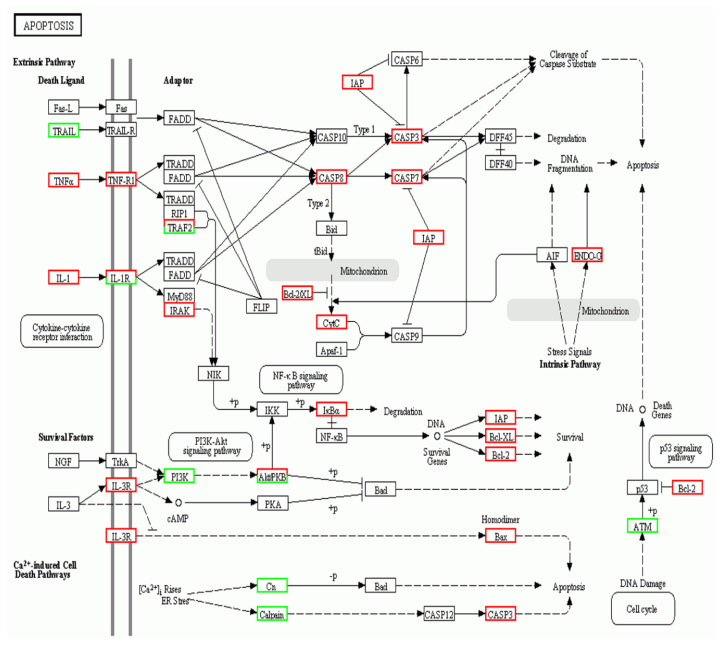
Significant differentially expressed genes identified by KEGG as involved in the apoptosis signaling pathway. Green boxes indicate significantly decreased expression. Red boxes indicate significantly increased expression. White boxes indicate unchanged expression.

To investigate the potential biological function of DEGs, GO, and KEGG analyses were executed for all DEGs. GO analysis found that all DEGs could be classified into 52 groups and three categories; biological process (21), cellular component (20), and molecular function (11) (Figure 3). A total of 663 DEGs were categorized into “response to stimulus”, including 199 up-regulated and 464 down-regulated DEGs. There were 114 (including 56 up-regulated and 58 down-regulated DEGs) in the “immune system process”, 493 (131 up-regulated and 362 down-regulated DEGs) in “signaling”, and 1131 (307 up-regulated and 824 down-regulated DEGs) in “biological regulation”. KEGG analysis found a total of 2084 DEGs were enriched into 192 pathways, the top 20 of which are shown in Figure 4. In particular, 14 immune-related pathways and one apoptosis-related pathway were screened including 127 DEGs (6.31%) in “cytokine-cytokine receptor interaction”, 82 (3.93%) in “phagosome”, and 43 (2.06%) in “toll-like receptor signaling pathway” (Table 3). There were 37 significant DEGs (including 22 up-regulated and 15 down-regulated) involved in the apoptosis-related signaling pathway. These DEGs are represented in the pathway diagram in different colors (Figure 5).

### 2.3. RT-qPCR Validation of DEGs

**Figure 6:**
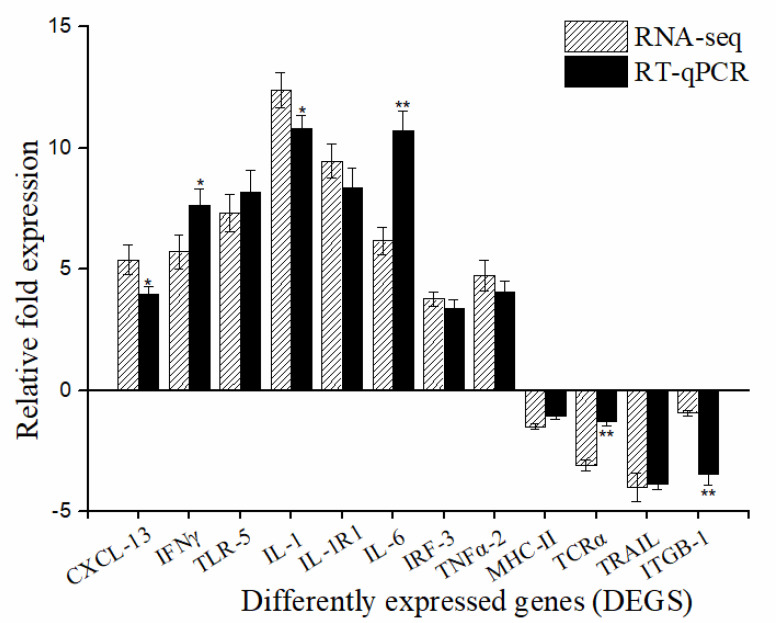
Comparison of the fold change expression of twelve putative differentially expressed genes (DEGs) between RNA-seq and qRT-PCR. The expression levels of selected genes by qRT-PCR were each normalized to that of the *β-actin* gene. The relative expression levels from the RNA-seq analysis were calculated as RPKM values. Each value is the mean of three samples. Bars represent mean ± S.E. ** *p* < 0.01; * *p* < 0.05.

Transcriptome and RNA-seq data were verified with 12 randomly selected DEGs for RT-qPCR analysis. These DEGs were chemokine (C-X-C motif) 13 (*CXCL-13*), interferon-gamma (*IFNγ*), toll-like receptor 5 (*TLR-5*), interleukin-1 (*IL-1*)*,* interleukin-1 receptor1 (*IL-1R1*), interleukin-6 (*IL-6*), interferon regulatory factor-3 (*IRF-3*), tumor necrosis factor alpha2 (*TNFα-2*), major histocompatibility complex class II (*MHC-II)*, T cell receptor alpha (*TCRα*), TNF-related apoptosis-inducing ligand (*TRAIL*), and integrin subunit beta 1 (*ITGB-1*). As shown in Figure 6, the fold-change values by RT-qPCR were consistent with the values obtained by RNA-seq for all selected genes.

### 2.4. Detection of Immune-Related DEGs after Challenge with A. schubertii

**Figure 7:**
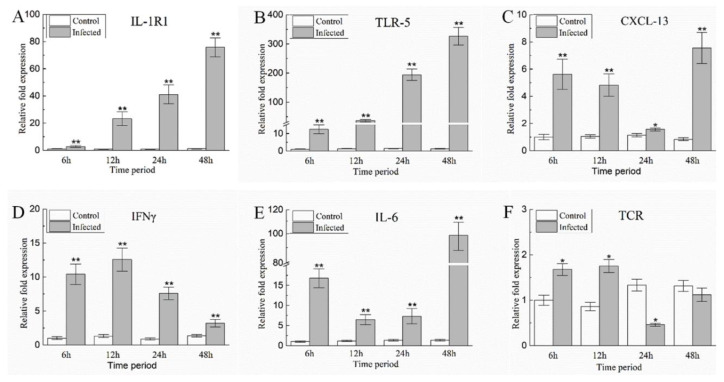
The relative expression levels of immune-related genes in the spleen of hybrid snakehead after infection with *A. schubertii*. Gene expression was analyzed by qRT-PCR with the 2^−^^ΔΔ^^Ct^ method, using *β-actin* as a reference. (**A**) *IL-1R1*, (**B**) *TLR-5*, (**C**) *CXCL-13*, (**D**) *IFNγ*, (**E**) *IL-6*, (**F**) *TCRα*. Each value is the mean of nine samples. Bars represent mean ± S.E. ** *p* < 0.01; * *p* < 0.05.

To further assess immune-related DEGs, hybrid snakeheads were challenged with *A. schubertii* and mRNA expression levels of six immune-related genes were examined at multiple time points post-infection (6, 12, 24, and 48 h). The results are shown in Figure 7. Gene expression levels are roughly clustered into two patterns. The most common pattern was displayed by five genes; *IL-1R1*, *TLR-5*, *CXCL-13*, *IFNγ*, and *IL-6*. The mRNA levels of these genes were significantly higher than control (*p* < 0.01) at 6, 12, 24, and 48 h after infection. A second pattern was observed for *TCRα*, which were significantly up-regulated at 6 and 12 h following infection, then decreased at 24 h, but returned to a near-normal level by 48 h.

### 2.5. A. schubertii Infection Induced Apoptosis in Hybrid snakehead

**Figure 8:**
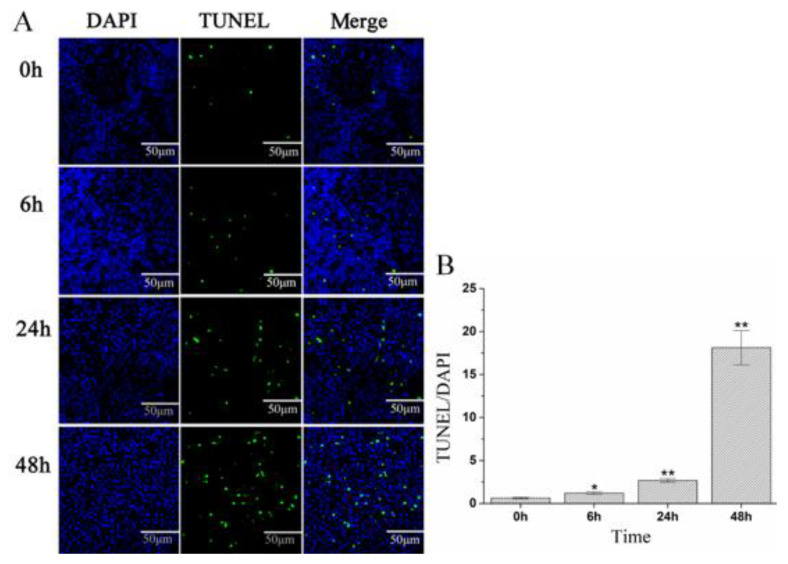
*A. schubertii* infection-induced apoptosis in spleens of hybrid snakehead at 0, 6, 24, and 48 h post-infection. Splenic *tissue sections* were analyzed by TUNEL staining. Apoptotic cells are stained *green* and *nuclei are* stained blue (**A**). The apoptotic cell rate during 0–48 h post-infection (**B**). Each value is the mean of three samples. Bars represent mean ± S.E. ** *p* < 0.01; * *p* < 0.05.

**Figure 9:**
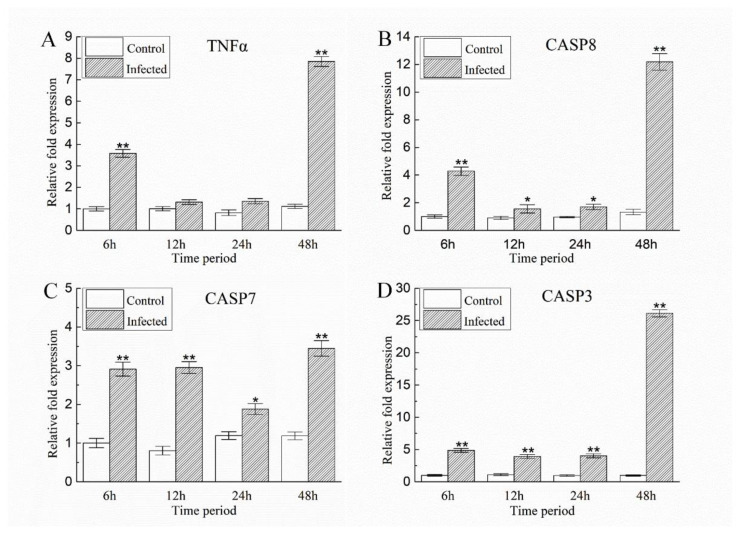
The relative expression levels of apoptosis-related genes in the spleen of hybrid snakehead after infection with *A. schubertii*. Gene expression was analyzed by qRT-PCR with the 2^−^^ΔΔCt^ method, using *β-actin* as a reference. (**A**) *TNFα*, (**B**) *CASP8*, (**C**) *CASP7*, (**D**) *CASP3*. Each value is the mean of nine samples. Bars represent mean ± S.E. ** *p* < 0.01; * *p* < 0.05.

Based on KEGG enrichment pathways analysis, *A. schubertii* infection significantly activated the cell apoptosis pathway. A TUNEL apoptosis detection kit was used to assess apoptosis of hybrid snakehead splenocytes at 0, 6, 24, and 48 h after *A. schubertii* infection. The TUNEL assay attaches green fluorescein to fragmented DNA, indicating an apoptotic cell. Nuclei have a blue fluorescent signal when labeled with DAPI. The number of green apoptotic cells in the spleen gradually increased with infection (Figure 8A). The apoptotic cell rate significantly increased 0–48 h post-infection. The apoptotic cell rate at 48 h (18.12) was more than 28 times greater than at 0 h (0.63) (Figure 8B). To further investigate apoptosis, the expression of four apoptosis-related genes (*TNFα, CASP3, CASP7,* and *CASP8*) was measured following infection by RT-qPCR. As shown in Figure 9, mRNA levels of *CASP3*, *CASP7,* and *CASP8* were significantly up-regulated compared to control (*p* < 0.05) at 6, 12, 24, and 48 h post-infection. *TNFα* mRNA levels were significantly up-regulated (*p* < 0.05) at 6 h, decreasing to nearly normal levels at 12 and 24 h, and then significantly up-regulated at 48 h.

## 3. Discussion

For the snakehead fish aquaculture industry, *A. schubertii* is an important pathogen. The infection produces typical nodules throughout the internal organs of diseased fish. These symptoms rarely occur with other Aeromonas infections. This observation implied that *A. schubertii* pathogenesis was distinct from common Aeromonas species [9]. Nodule formation has traditionally been considered a dangerous strategy by which to limit infection, protecting the host by walling off resistant pathogens [33,34]. However, some nodules (such as granuloma caused by pathogenic mycobacteria) paradoxically allow the pathogens to multiply and disseminate infection, promoting disease progression [35,36]. In our previous studies, we reported similar nodules in tilapia infected with *A.* *schubertii*. Histopathological analysis showed the center of the nodules to contain collections of bacteria surrounded by degenerate and necrotic tissue, which is similar to the granuloma caused by mycobacteria [9]. Few studies have reported interactions between *A. schubertii* and hybrid snakehead. Furthermore, the host immune response to *A. schubertii* during infection is still largely unknown.

Transcriptome analysis has proven to be a useful tool by which to elucidate pathogenic mechanisms. Herein, we established an *A. schubertii* infection model in juvenile hybrid snakeheads. The clinical signs of the challenged fish were similar to those already described for other *A. schubertii* infected fish such as snakehead and tilapia [6,9,37]. Sequencing of the splenic transcriptome response of hybrid snakehead with or without *A. schubertii* infection allowed for analysis of the pathogen-host interaction during infection. In total, 14,796 DEGs including 4441 (accounting for 30.01% of all DEGs) up-regulated genes and 10,355 (accounting for 69.99%) down-regulated genes were found to differ between groups. This number is greater than the number found in peripheral blood of hybrid snakehead after *Nocardia seriolae* infection [38], or in dissected muscle tissue of *C. striatus* after *Aphanomyces invadans* infection [39]. The spleen is an important immune organ in fish. Previous comparative transcriptome analysis of hybrid sturgeon (*Huso dauricus × Acipenser schrenckii*) spleens identified 283 DEGs (168 up-regulated genes and 115 down-regulated genes) between *A. hydrophila* infected fish and control. Various anti-bacterial immune-relevant pathways were identified [40]. Therefore, we focused on DEGs related to the splenic immune response after infection. Herein, functional enrichment analysis showed 371 DEGs (196 up-regulated genes and 175 down-regulated genes) in 14 immune-related KEGG pathways that were closely involved in anti-bacterial immune responses, indicating a complicated immune system in hybrid snakeheads.

The innate immune system operates the first line of defense against invading pathogens by potential pathogen recognition [41]. Pattern recognition receptors (PRRs) recognize microbial pathogen-associated molecular patterns (PAMPs) that trigger the innate immune system [42]. The major families of PRRs are the Toll-like receptors (TLRs), nucleotide-binding and oligomerization domain (NOD)-like receptors (NLRs), and retinoic acid-inducible gene I (RIG-I)-like receptors (RLRs). In vivo challenge experiments in teleost species have shown tissue activation of PRR signaling pathways following bacterial infection [40,43,44]. Herein, we observed the number of up-regulated DEGs to be greater than those down-regulated in the TLR signaling pathway, the RIG-I-like receptor signaling pathway, the NLR signaling pathway, and the cytosolic DNA-sensing pathway (Table 3). Furthermore, the time course RT-qPCR analysis demonstrated *A. schubertii* infection to progressively increase mRNA levels of two PRRs (*TLR-5* and *IL-1R1*). TLR-5 is known to recognize bacterial flagellin of invading bacteria, resulting in activation of the key NF-κB signaling pathway that leads to proinflammatory gene program activation [45]. *IL-1R1* recognizes and binds to the extracellular domain of *IL-1* related cytokines that are involved in multiple immunological and inflammatory processes [46]. The time course RT-qPCR analysis demonstrated a dramatic increase in the expression of the proinflammatory cytokine mRNAs for *CXCL-13*, *IFNγ*, and *IL-6* with infection. These cytokine genes have been associated with the innate immune response, playing an important role in defense against pathogenic microorganisms in many species of fish [47,48,49]. Therefore, their up-regulation indicated that PRRs and the related proinflammatory response may represent an important anti-infection mechanism during the early stage of *A. schubertii* infection. In addition, transcriptome analysis identified a small number of DEGs in other immune-related signaling pathways including Fc gamma R-mediated phagocytosis (two down-regulated genes), B cell receptor signaling pathway (two down-regulated genes), natural killer cell-mediated cytotoxicity (two down-regulated genes), Fc epsilon RI signaling pathway (two down-regulated genes), hematopoietic cell lineage (two down-regulated genes), and complement and coagulation cascades (one up-regulated gene). These results are inconsistent with other studies of bacterial infections [41,50] but are partially consistent with the analysis of grass carp intestines following infection with *A. hydrophila* [12]. Thus, we propose *A. schubertii* may have evolved highly sophisticated mechanisms to modulate host immune responses that ensure successful infection.

Apoptosis is a primary form of programmed cell death that plays an important role in the regulation of growth, development, and immune responsiveness [51]. Oligo Nucleosomal DNA fragmentation and chromatin condensation are two hallmarks of apoptosis [52]. The TUNEL technique can sensitively detect DNA fragmentation and is a standard means by which to detect apoptosis in tissue sections [53]. TUNEL has been widely used to detect apoptotic cells during pathogen invasion. Pathogenic *bacteria* known to trigger host cell apoptosis are *A. hydrophila* [54], *Mycoplasma gallisepticum* [55], and *Mycobacterium tuberculosis* [56]. In a previous study, TUNEL found 50 to 70% apoptotic cells in the periphery of mycobacterial granulomas in clinical cases of tuberculosis, which implied that apoptosis may be involved in the formation of granulomas [57]. The major histological feature of *A. schubertii* infection is the nodule, which has a structure similar to that of mycobacterial granulomas [9]. In this study, TUNEL demonstrated the rate of apoptosis to significantly increase in the spleens of fish after *A. schubertii* infection, reaching 18.12 at 48 h. This result showed that prolonged infection induced severe cellular apoptosis. We speculate that *A. schubertii*-induced apoptosis is an important cause of nodule formation in hybrid snakeheads.

As an indispensable defense mechanism for host resistance to pathogen invasion, apoptosis mainly occurs in three basic ways: death receptor, endoplasmic reticulum, and mitochondrial pathways [58,59]. The signaling pathways (intrinsic or extrinsic) of apoptosis are complex but always rely on the activation of caspases (cysteine proteases) [60]. In this study, transcriptome analysis demonstrated apoptosis-related pathway molecules to be involved in *A. schubertii* infection (Figure 5). For example, death receptor pathway-related genes: *TNFα*, *TNF-R1*, *TRAF2*, *IRAK*, *CAPS8*, *IAP*, *CASP3,* and *CASP7* were up-regulated, while *TRAIL* was down-regulated, suggesting that the death receptor signaling pathway may be crucial to *A. schubertii* infection. The expression of *TNFα*, *CASP8*, *CASP7,* and *CASP3* genes was assessed at 6, 12, 24, and 48 h post *A. schubertii* challenge. TNFα induces CASP8-dependent apoptosis through the formation of a death-inducing signaling complex comprised of the TNF receptor-associated death domain (TRADD) and the death domain (FADD) [61]. CASP8 is a cysteine protease best known for mediating the downstream caspase (CASP 3, 6, 7) death receptor signaling pathway that results in apoptosis [61,62]. CASP8, CASP 7, and CASP3 are thought to be key factors in the caspase cascade of apoptosis execution by cleaving multiple structural and repair proteins [63]. Herein, the expression of CASP8, CASP7, and CASP 3 was significantly up-regulated after infection, with the expression of TNFα significantly up-regulated at 6 and 24 h post-infection. This observation suggested that *A. schubertii* infection activated the TNFα-related death receptor apoptosis pathway in hybrid snakeheads.

## 4. Materials and Methods

### 4.1. Fish Sample, Bacteria Preparation, and Ethics

Healthy juvenile hybrid snakeheads weighing 30 ± 2 g were obtained from a local fish farm in Guangzhou City, Guangdong Province, China. Fish were kept in aerated tap water at 28 °C with commercial feed for 2 weeks. The bacterial strain of *A. schubertii* used for the experiments was isolated from a diseased snakehead fish [8]. Bacteria were cultured in Brain-Heart infusion agar (BHIA) at 28 °C for 12 h with constant moderate shaking (180 rpm), then harvested by centrifugation at 4000× *g* for 10 min, washed once with phosphate-buffered saline (PBS, pH 7.2), and centrifuged (4000× *g*, 10 min). Bacterial pellets were resuspended in PBS and the concentration adjusted.

### 4.2. Bacterial Challenge, Sample Collection, and Total RNA Extraction

To collect samples for transcriptome sequencing, ten healthy snakeheads (water temperature at 28 ± 2 °C) were injected intraperitoneally with a suspension of *A. schubertii* (3 × 10^6^ CFU/fish). Ten fish treated with 0.2 mL of PBS were used as control. Eight h post-infection, three fish from each group were anesthetized with MS-222 and their spleens collected (SBT-1, SBT-2, and SBT-3 from the *A. schubertii* infected group) and (NC-1, NC-2, and NC-3 from the control group) for RNA-seq. The samples were stored in liquid nitrogen until RNA extraction. The remaining fish were continuously monitored for clinical signs until 7 days post-infection.

To investigate fish apoptosis and immune response, 120 healthy snakeheads were randomly divided into two groups. Sixty test fish (water temperature 28 ± 2 °C) were intraperitoneally inoculated with *A. schubertii* (3 × 10^5^ CFU/fish). Another sixty fish were treated with 0.2 mL of PBS as control. In genes expression experiments, nine fish from each group were anesthetized with MS-222, and spleen samples were obtained at 6, 12, 24, and 48 h post-infection. Each sample consisted of a mixture of tissues from three fish. Three replicates of each sample were assessed for each time point. All samples were frozen in liquid nitrogen until RNA extraction. For apoptosis experiments, three fish from the infected group were anesthetized with MS-222, and spleen samples were taken at 0, 6, 24, and 48 h post-infection for analysis by the TUNEL assay.

Total RNA was extracted using TRIzol Reagent (Invitrogen, Carlsbad, CA, USA). The mRNA was reverse-transcribed using PrimeScript RT reagent Kit with gDNA Eraser (Takara, Dalian, China), following the manufacturer’s recommendations.

### 4.3. Library Preparation and RNA-seq

Total RNA quality was examined using an Agilent 2100 Bioanalyzer (Agilent Technologies, Palo Alto, CA, USA). RNA-seq was conducted using an Illumina HiSeq 4000 platform (Gene Denovo, China). Briefly, mRNA was purified using oligo (dT) magnetic beads and fragmented into short fragments of 300–400 nucleotides using an RNA fragmentation reagent (Thermo Fisher Scientific, San Francisco, CA, USA). Subsequently, 3’ and 5’ adaptors were ligated into unique RNA fractions, adaptor-ligated RNA fragments were reverse-transcribed and amplified by qPCR, and then the RNA library was sequenced. RNA-seq raw data have been submitted to the NCBI SRA database and the accession number is SRP108948.

### 4.4. Transcriptome Assembly and Unigene Annotation

Raw Illumina paired-end reads were filtered using internal software to remove reads without adaptors; those reads in which unknown bases (N) comprised greater than 5% of the read, and low quality reads. Post-filtered reads (clean reads) were stored in FASTQ format. Trinity was used to perform de novo assembly with clean reads. Next, TGICL gene indices clustering tools were used to cluster transcripts into unigenes. All unigenes were annotated with the following databases: Nr (NCBI non-redundant protein database), Swiss-Prot, KOG (Clusters of Orthologous Groups), and KEGG (Kyoto Encyclopaedia of Genes and Genomes) with E-values less than 10^−5^.

### 4.5. Enrichment Analysis of DEGs

Expression data from two libraries (NC and SBT) were determined by mapping to the transcriptome assembly using Bowtie 2 software. The reads per kb of transcripts per million fragments mapped (RPKM) values were analyzed using RESM and differentially expressed genes were obtained using edgeR NC and SBT spleens. Furthermore, to determine the threshold *p*-value in multiple tests, a false discovery rate (FDR) was used. Significant enrichment was calculated when FDR was <0.05 and RPKM values showed at least a two-fold difference between the two samples. GO was performed to identify the functional annotations of the DEGs. Goatools software (Available online: https://github.com/tanghaibao/Goatools, accessed on 13 September 2017) was used to analyze the functional enrichment terms. Significant enrichment was considered when FDR was <0.05. We also used the KEGG Orthology Based Annotation System (KOBAS; Available online: http://kobas.cbi.pku.edu.cn/home.do, accessed on 15 September 2017) to perform the KEGG pathway analysis, with enriched pathways determined by hypergeometric test (filtered with FDR < 0.05).

### 4.6. Real-Time Fluorescence Quantitative PCR (RT-qPCR) Analysis

To validate the RNA-seq data, 12 randomly selected genes were chosen from all of the DEGs. The mRNA expression levels of these selected genes were then determined by RT-qPCR. RT-qPCR analysis was performed as described previously [64]. RT-qPCR was performed using an SYBR Premix Ex Taq kit (Takara, Dalian, China) with a QuantStudio 7 Flex Real-Time machine (Applied Biosystems, Waltham, MA, USA). All reactions were performed in triplicate. Relative expression levels of mRNAs were calculated using the 2^−^^ΔΔCt^ method. *β-actin* was used as a housekeeping gene for the normalization of mRNA.

The same procedure was used to measure the expression profiles of 10 selected genes involved in immune response and apoptosis pathways (KEGG pathway: hsa04210). The oligonucleotide primer pairs used for all RT-qPCR reactions are listed in Table S1.

### 4.7. Apoptosis Analysis

Apoptosis was evaluated using TUNEL staining. Hybrid snakeheads were infected with *A. schubertii* as described above. After 0, 6, 24, and 48 h post-infection, spleens were fixed in 4% (*w/v*) paraformaldehyde for at least 24 h, dehydrated through a graded alcohol series to xylene, embedded in paraffin wax, and sectioned at 3–5 μm. The spleen tissue sections were subjected to TUNEL staining with a TUNEL assay kit (Roche, Switzerland) as described previously [65]. After staining, the sections were viewed by Ortho-Fluorescent Microscopy (Nikon Eclipse C1, Tokyo, Japan) with cell apoptosis and apoptosis rate judged by Image-pro plus 6.0 (Media Cybernetics, Bethesda, MD, USA) Three fields were examined for each sample and statistically analyzed.

### 4.8. Statistical Analysis

Statistical analysis was performed using SPSS 19.0 software (SPSS Inc., Chicago, IL, USA). All data are presented as means and standard error. One-way ANOVA and Duncan’s *t*-tests were used to analyze the data. The minimum level of significance level was set as *p* < 0.05.

## 5. Conclusions

In conclusion, we have shown that *A. schubertii* infection induced sophisticated immunological reactions in hybrid snakeheads. These reactions included up-regulation of cytokine genes, increased expression of PRRs, a pro-inflammatory response, and activation of the death receptor apoptosis pathway. Our results provided valuable information about the immune response of hybrid snakeheads during the early stages of a bacterial infection. Furthermore, these findings provided new insights into anti-*A. schubertii* immunity in hybrid snakehead, which can be used to unravel *A. schubertii* infections in fish at the molecular level.

## Figures and Tables

**Table 1:** Overview of RNA-seq.

Sample	Raw Reads	Clean Reads	Clean Base	GC
NC-1	33,306,402	32,889,174	4.92G	47.02%
NC-2	33,820,162	33,376,306	4.99G	46.84%
NC-3	36,227,606	35,749,772	5.34G	47.11%
NC-total	103,354,170	102,015,252	15.25G	46.99%
SBT-1	30,868,936	30,479,570	4.57G	46.76%
SBT-2	29,255,268	28,832,816	4.32G	46.49%
SBT-3	31,578,840	31,111,042	4.67G	47.12%
SBT-total	91,703,044	90,423,428	14.55G	46.79%

Note: NC and SBT represent the control and infection groups, respectively.

**Table 2:** Summary annotations of assembled unigenes.

Database	Annotated Unigenes	Percentage
Nr	26,981	40.95%
Swiss-Prot	21,484	32.61%
GO	12,786	19.40%
KEGG	15,001	22.77%
KOG	16,385	24.87%
All databases	11,809	17.92%
At least one database	27,533	41.79%
Total unigenes	65,884	100%

**Table 3:** Immune-related and apoptosis pathways identified after *A. schubertii* infection.

Pathway ID	KEGG Pathways	DEGs		Total
		Up	Down	
ko04060	Cytokine-cytokine receptor interaction	67	66	133 (6.38%)
ko04145	Phagosome	33	49	82 (3.93%)
ko04620	Toll-like receptor signaling pathway	33	10	43 (2.06%)
ko04672	Intestinal immune network for IgA production	12	22	34 (1.63%)
ko04622	RIG-I-like receptor signaling pathway	19	8	27 (1.30%)
ko04621	NOD-like receptor signaling pathway	13	8	21 (1.01%)
ko04623	Cytosolic DNA-sensing pathway	16	1	17 (0.82%)
ko04062	Chemokine signaling pathway	2	1	3 (0.14%)
ko04666	Fc gamma R-mediated phagocytosis	0	2	2 (0.10%)
ko04662	B cell receptor signaling pathway	0	2	2 (0.10%)
ko04650	Natural killer cell-mediated cytotoxicity	0	2	2 (0.10%)
ko04664	Fc epsilon RI signaling pathway	0	2	2 (0.10%)
ko04640	Hematopoietic cell lineage	0	2	2 (0.10%)
ko04610	Complement and coagulation cascades	1	0	1 (0.05%)
ko04210	Apoptosis	22	15	37 (1.78%)

## Data Availability

The raw data reads were deposited in NCBI SRA database (https://www.ncbi.nlm.nih.gov/sra, accessed on 6 August 2021) under the accession number SRP108948.

## Supplementary Materials

The following are available online at https://www.mdpi.com/article/10.3390/pathogens10080997/s1, Table S1. Primers used for qPCR in this study.
