# Supplementary material for: Immune Response and Apoptosis-Related Pathways Induced by Aeromonas schubertii Infection of Hybrid Snakehead (Channa maculata♀ × Channa argus♂)
Source: Pathogens. 2021 Aug 7;10(8):997. doi: 10.3390/pathogens10080997 (PMC8401259; doi:10.3390/pathogens10080997)
Supplement: Supplementary file 1 [file pathogens-10-00997-s001.zip › pathogens-1248118-supplementary.pdf]

Table S1. Primers used for qPCR in this study.

Primer Name	Sequence (5'-3')	length (bp)
CXC-13F	TTTGGACCATTGCTTTGCG	151
CXC-13R	TGCTCCTGGCTGCTCTGACT	
IFNγF	CTCTGGCTGGCTGTGTATCG	182
IFNγR	GCAGCAGGTTCTGGATGGTT	
ITGB1F	CCAGAGGGAGTGACCATAGCGT	138
ITGB1R	GCATTTCCGTCCATTTTCGC	
IL-1F	TAGTGCCACAGGTCGGTCGT	165
IL-1R	GCGAATCAACTCCTTCTGCTC	
IL-1R1F	ACTCAGCGTGCTCATCAAAGA	188
IL-1R1R	GTGGTGAAACAATCAAAGGTGG	
IL-6F	GGTGATGAGGAGGTGGAGCC	201
IL-6R	GAGGCAGGACATCTTGGTGG	
IRF3F	CCCAAGAGATTTCTAAACTGCCAC	144
IRF3R	CGACCTCCACTGTGATAAGTTTCT	
MHCIIF	TTTCTGTGAGTGAGCGGGAGC	157
MHCIIR	GGAGCAGGATAGAAACCAGTCACAT	
TCRF	GCTGGTCATAGTCACAAAGTTCAC	163
TCRR	TACAAAGTGACAGGTGATGATGGG	
TLR-5F	GAAACAAGCCTTCGTGGGACT	171
TLR-5R	GAGGACAGTGGCTCCAGATAGTT	
TRAILF	CCTTCGCTTGTGATGGATGAC	184
TRAILR	TCTCGGTCCACTTTGGGCAT	
TNFa2F	TCCTCACAAGGCAAAGACACC	182
TNFa2R	CAGATGAACACGGGAGATGG	
β-actinF	CAATGAGAGGTTCAGGTGCCCAGAG	186
β-actinR	AGTGTTGGCATACAGGTCCTTACGG	
Casp3F	GCACTATTTCCGTGGTCCTCCTT	164
Casp3R	ATGGGACTGATGTTGATGCCG	
Casp7F	CCTCTTTGCCTACTCCACTGTTCC	159
Casp7R	GCATAGAAACCACGCAGGGAAT	
Casp8F	CATCAACCAGCACCACCTTACCT	134
Casp8R	ACCACACACCAACCACGAGGAT	
TNFaF	GGACACAGTCAGGCAGATTGGAT	172
TNFaR	TGTTTTGTTGGTTCTGGTGGTTGT	
